# Electrical Imaging of Light-Induced Signals Across and Within Retinal Layers

**DOI:** 10.3389/fnins.2020.563964

**Published:** 2020-11-19

**Authors:** Meng-Jung Lee, Günther Zeck

**Affiliations:** ^1^Neurophysics, NMI Natural and Medical Sciences Institute at the University Tübingen, Reutlingen, Germany; ^2^Graduate School of Neural Information Processing, International Max Planck Research School, Tübingen, Germany

**Keywords:** retina, retinal slice, CMOS-MEA, microelectrode array, electrical imaging

## Abstract

The mammalian retina processes sensory signals through two major pathways: a vertical excitatory pathway, which involves photoreceptors, bipolar cells, and ganglion cells, and a horizontal inhibitory pathway, which involves horizontal cells, and amacrine cells. This concept explains the generation of an excitatory center—inhibitory surround sensory receptive fields—but fails to explain the modulation of the retinal output by stimuli outside the receptive field. Electrical imaging of light-induced signal propagation at high spatial and temporal resolution across and within different retinal layers might reveal mechanisms and circuits involved in the remote modulation of the retinal output. Here we took advantage of a high-density complementary metal oxide semiconductor-based microelectrode array and investigated the light-induced propagation of local field potentials (LFPs) in vertical mouse retina slices. Surprisingly, the LFP propagation within the different retinal layers depends on stimulus duration and stimulus background. Application of the same spatially restricted light stimuli to flat-mounted retina induced ganglion cell activity at remote distances from the stimulus center. This effect disappeared if a global background was provided or if gap junctions were blocked. We hereby present a neurotechnological approach and demonstrated its application, in which electrical imaging evaluates stimulus-dependent signal processing across different neural layers.

## Introduction

In the mammalian retina, different morphological and functional types of retinal ganglion cells (RGCs) cover the visual space with their receptive field in a mosaic organization to encode the different features of visual scenes uniformly (Wassle, 2004; Field et al., 2007; Anishchenko et al., 2010). This mosaic-like structural and functional organization is guaranteed by the classical receptive field, which refers to the region in visual space driving neuronal activity (i.e., RGC action potentials). At the excitatory receptive field center, light is captured by photoreceptors (PRs), further transduced to bipolar cells (BCs), and turned into spiking output through RGCs by glutamate release in the vertical signal transduction pathways. The antagonistic surround, which suppresses the excitatory center, tunes the light response of BCs and RGCs *via* lateral inhibition in both the outer plexiform layer (OPL) and the inner plexiform layer (IPL) by horizontal cells and amacrine cells (ACs), respectively. While the surround usually serves as an inhibitory input to the center response, previous studies showed that there are exceptions.

As the size of stimulation increases, the inhibition strength could decrease instead of increase (Mani and Schwartz, 2017) or, furthermore, change the polarity of the RGCs (Kim et al., 2008). Other studies showed that visual stimulation at remote areas in visual space situated outside the classical receptive field of a RGC modulates its activities under certain conditions (Passaglia et al., 2001; Chiao and Masland, 2003; Olveczky et al., 2003; Passaglia et al., 2009; Deny et al., 2017). These non-classical activations involve the complex modulation of the lateral inhibition or disinhibition. In the mouse retina, at least 14 types of BCs (Behrens et al., 2016; Tsukamoto and Omi, 2017), more than 40 types of ACs (Diamond, 2017), and more than 30 types of RGCs (Baden et al., 2016) form a delicate visual network to process different visual features; any modification of connectivity between cells leads to activity change. Focusing on the stimulus-induced activity change in one or few very specific cell types reveals the microscopic circuitry and the underlying signal processing mechanisms; however, the macroscopic understanding of how the different retinal layers contribute to signal processing on a global scale remains elusive.

Fluorescence-based methods, which study both the vertical and the lateral network at the same time, go with the trade-off between recording area and time resolution (Lillis et al., 2008; Zhao et al., 2020). Here we propose a methodological approach by adapting a retinal vertical slice onto the high-density complementary metal oxide semiconductor-based microelectrode array (CMOS MEA; Bertotti et al., 2014) to study signal processing across different layers using electrical imaging (Zeck et al., 2017) over large areas (1 mm^2^) at high temporal (milliseconds) and spatial resolution (micrometer).

By imagining the propagation of local field potentials (LFP) in vertical retinal slices upon well-defined local light stimuli and comparing them to ganglion cell recordings in a flat-mount configuration, we were able to identify light conditions which stimulated remote RGCs and investigate potential mechanisms.

## Materials and Methods

### Animals and Retina Preparation

In this study, adult C57BL/6J mice aged between 1 and 7 months of either sex were used. All the animals were housed in a 12-h day–night rhythm. The mice were made to adapt in the dark for at least 1 h prior to the experiments, anesthetized with CO_2_, and euthanized by cervical dislocation. The eyes were removed and immediately transferred to carboxygenated (95% O_2_ and 5% CO_2_) Ames’ medium (23 mM NaHCO_3_, A1420, Merck KGaA; Sigma Aldrich). The isolated eyes were both hemisected first and then cut into half or 1/3 slices for the flat-mount preparation, respectively. The retina was carefully detached from the retinal pigment epithelium, and the vitreous body was removed from it. The whole procedure was performed at room temperature in carboxygenated Ames’ medium under dim red light (long-pass filter > 640 nm).

For the retinal slice preparation, a 35-mm petri dish was half-filled with 4% low-melting agarose gel (6351.5, Carl Roth, Germany), and we waited until it solidified. The retina was transferred to the top of the solidified low-melting agarose gel with the RGCs side up, then the excess Ames’ medium was removed to flatten the retina. At 37^°^C, 4% low-melting agarose gel was gently poured into the petri dish to embed the retina, and then the petri dish with the retina was immediately transferred on ice for 1 min for the solidification of the newly added gel. Afterward, the agarose gel block with the retina was trimmed into a proper size and glued onto the vibratome specimen disk with histoacryl (1050052, B. Braun, Germany). A similar size of 5% broad-range agarose gel block (T846.2, Carl Roth) was glued right next to the gel block with the retina at the opposite side of the blade from the vibratome as support from the force of slicing. The specimen disk was placed into the buffer tray and filled with cooled, carboxygenated Ames’ medium. The retina in the buffer tray was placed onto the vibratome (VT1200 S vibrating blade microtome, Leica), and the flat-mounted retina was sliced into 500-μm-thick slices with a razor blade (Extra Double Edge Safety Razor Blades, Derby), vibrating in 0.01 mm/s speed and 0.25 mm amplitude. The slices were kept in Ames’ medium in 37^°^C water bath with continuous carboxygenation until use. Details of the slice preparation and of the interfacing to the CMOS MEA are presented in Supplementary Figure 1.

All procedures were approved by the animal use committee of the Natural and Medical Science Institute at the University Tübingen and performed in compliance with the ARVO statement for the use of animals in ophthalmic and visual research. Protocols compliant with section “Discussion”, paragraph 3 of the German law on animal protection were reviewed and approved by the Regierungspräsidium Tübingen (AZ 35/9185.82-7). All efforts were made to minimize the number of animals.

### Recording of Retinal Tissues Using CMOS-Based Microelectrode Arrays

**FIGURE 1:**
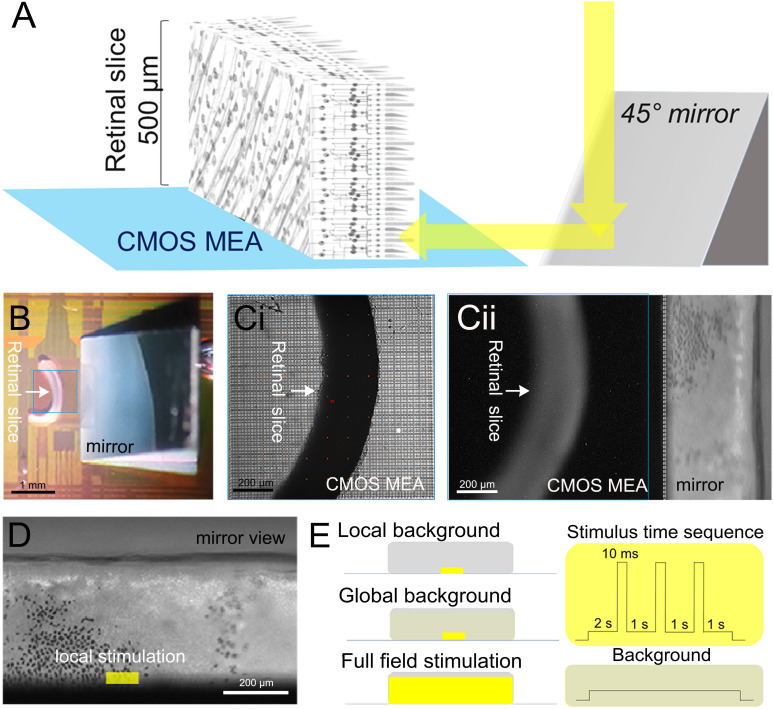
Experimental setup and light stimulation protocol. **(A)** Schematic description of the experiments investigating signal propagation in the retinal layers of a vertical slice. Light is projected through a microscope objective onto a 45° mirror then stimulated the photoreceptor layer. **(B)** Photograph showing the slice interfaced to the 1-mm^2^ sensor surface of a complementary metal oxide semiconductor-based microelectrode array (marked by a blue square). The mirror is visible on the right. **(C,i)** Photograph taken through the microscope objective focused on the sensor surface. **(C,ii)** Photograph taken through the microscope objective focused on the mirror. The photoreceptor layer with scattered black retinal pigment epithelial cells (black spots) is visible. **(D)** Zoomed image onto the micro-mirror. The overlaid light stimulus area is 100 × 30 μm^2^. **(E)** Description of the three light stimuli: local background stimulus: 100 × 30 μm^2^ light stimulus on a local background (100 × 30 μm^2^); global background stimulation: 100 × 30 μm^2^ light stimulus on a global background (1,000 × 300 μm^2^); and full-field stimulus: 1,000 × 300 μm^2^ light stimulus on a global background (1,000 × 300 μm^2^). On the right-hand side, the time sequence common to the three light stimuli with variable duration (10–320 ms) interleaved by 1-s-long background stimuli is shown (for details, see “Materials and Methods” section).

The sensor area (4,225 electrodes; electrode diameter, 8 μm; electrode spacing, 16 μm; size of the sensor area, 1 mm^2^) of a high-density CMOS-based MEA (CMOS MEA 5000-System, Multi-Channel Systems MCS GmbH, Reutlingen, Germany) was used to record the retinal activity. The CMOS MEAs were first cleaned with 80^°^C Tickopur R60 (Dr. H. Stamm GmbH, Germany). For the retinal slice preparation, after rinsing with bidistilled water, a 3-mm right-angle mirror (#49-405, Edmund Optics, Germany) was adapted onto the CMOS MEA outside the recording area (Figures 1A,B). The right-angle mirror was preprocessed with a 20-μm-thick silicone layer (SILPURAN^®^ Film 2030, Wacker Chemie AG, Germany) attached to the bottom so that the mirror can stick to the surface of the CMOS MEA. The CMOS MEA surface is then coated with poly-L-lysine hydrobromide solution until used (1 mg/ml in bidistilled water, 150 kDa molecular weight; Sigma Aldrich, Germany). Prior to retinal interfacing, the CMOS MEA was rinsed with Ames’ medium. A retinal slice was placed onto the coated CMOS MEA with the cut side, and a small amount (30–50 μl) of 4% low-melting agarose gel was dropped on top of the positioned retinal slice and allowed to solidify to ensure the position of the retinal slice during the whole recording (Figure 1). For the flat-mount recordings, the CMOS MEAs were cleaned and coated as mentioned above. After removal of the vitreous body, the retina was placed RGC side down onto the CMOS MEA. The retinas were constantly perfused with carboxygenated Ames’ medium at 33–35^°^C. Both the retinal slice and the flat-mount preparation were kept in the recording chamber for at least 30 min before the beginning of recording to ensure stable neural activity. The retinal activities were recorded with 20 kHz sampling rate.

### Optical Stimulation

In the retinal slice preparation, we first located the relative position of the slice and of the mirror under a dim red light, and then we adjusted the focus to acquire the image of the PR layer reflected from the mirror (Figure 1C). The light stimulation areas were selected precisely using the μ-Matrix system (Rapp OptoElectronic GmbH, Germany), and the light stimulus was projected onto the PRs *via* the mirror (Figure 1D).

The light stimulation protocol is schematically shown in Figure 1E. Light pulses (490 nm) were provided by an LED light source (CoolLED Ltd., Andover, United Kingdom) projected onto a digital micromirror device (Rapp OptoElectronic GmbH, Germany). After 2 s of adaptation, short light pulses of 10, 20, 40, 80, 160, and 320 ms were given onto the PR layer.

Combinations from two stimulus sizes (small field: 100 × 30 μm^2^ and global field: 1,000 × 300 μm^2^) and two light intensities (light stimulus: 10^7^ R^∗^ rod-1 s-1 and background: 10^5^ R^∗^ rod-1 s-1) form the three stimuli used for this study: (1) local background: a small field light stimulus projected onto the same size of local background, (2) global background: a small field light stimulus projected onto a global background, and (3) full field stimulus: global field stimulation projected onto a global field background (Figure 1E). For the flat-mount stimulation, the same three stimuli were applied directly without a mirror to the PRs through a ×5 objective.

### Pharmacological Treatment

The drug solution was carboxygenated, and bath was applied through perfusion for at least 15 min before the recordings. We used 100 μM meclofenamic acid (MFA; M4531, Sigma-Aldrich) to block gap junctional coupling. In additional experiments (Supplementary Figures), the following drug concentrations were used (in μM): 50 6,7-dinitroquinoxaline-2,3-dione (DNQX, 0189, TOCRIS, Bristol, United Kingdom) and 50 DL-2-amino-5-phosphonopentanoic acid sodium salt (DL-AP5, 3693, TOCRIS) to block ionotrophic glutamate receptors and 50 1,2,5,6-tetrahydropyridin-4-yl methylphosphinic acid (TPMPA, 1040, TOCRIS) and 25 6-Imino-3-(4-methoxyphenyl)-1(6H)-pyridazinebutanoic acid hydrobromide (SR95531, 1262, TOCRIS) to block GABA receptors.

### Data Analysis—Vertical Retinal Slices

#### Reconstruction of Electrical Images

**FIGURE 2:**
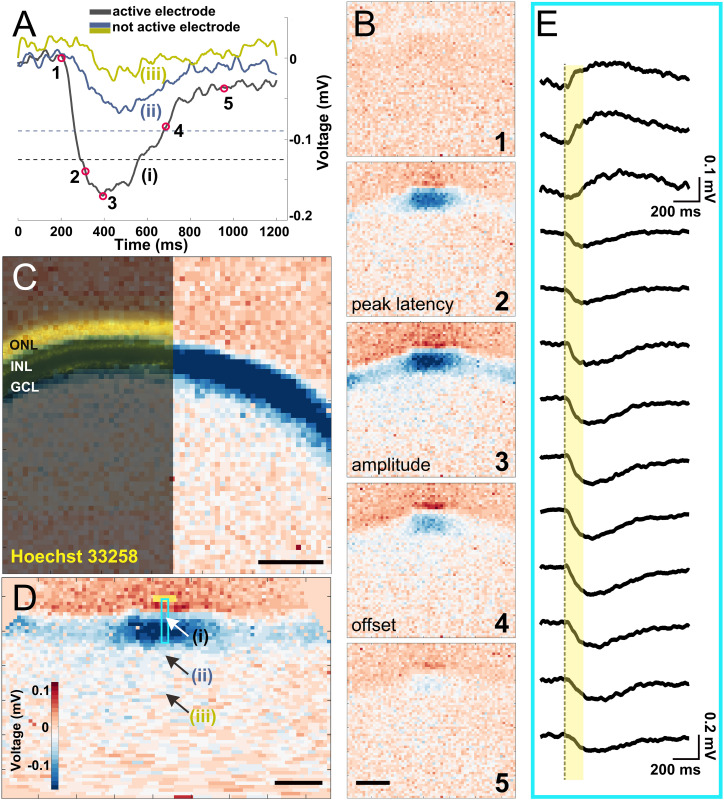
Electrical imaging the light-induced activity in a vertical slice. **(A)** Voltage traces of light-induced activity from a 160 ms long stimulus on a local background. Three recorded traces from electrodes representing an (i) active electrode whose amplitude crosses the threshold (black dashed line), (ii) inactive electrode which detected depolarizing signals with an amplitude below the threshold (blue dashed line), and (iii) an inactive electrode ∼250 μm from the slice. The electrode positions (i–iii) are marked in **(D)**. **(B)** Exemplary electrical images at five timepoints marked in **(A)**. The timepoints of 2, 3, and 4 were used in further analysis for peak latency, amplitude, and offset latency, respectively. **(C)** Overlay of Hoechst 33258 nuclear dye staining with an electrical image obtained after full-field light stimulus (160 ms duration). Nuclear dye staining revealed the three major cell layers (outer nuclear layer, inner nuclear layer, and ganglion cell layer) that matched the physiological recording. **(D)** The reconstructed straightened vertical slice (see “Materials and Methods” section) of an electrical image from timepoint 3 in **(B)**. The three arrows point to the electrode traces shown in **(A)**. The light blue rectangle marks the electrodes shown in detail in **(E)** upon stimulation with a light stimulus (yellow box). **(E)** Extracellular voltage recorded by 13 electrodes arranged along one column under the slice [marked by a light blue rectangle in **(D)**], covering a distance of ∼200 μm. The yellow bar indicates light stimulus duration. Scale bars in **(B–D)**: 200 μm.

Each dataset was averaged using 12 repeats of the stimulus and afterward smoothened by third-degree Savitzky–Golay filter. For each slice sample, the light response (electrical image, i.e., Figure 2B) from 160-ms full-field stimulus was used as a standard image for fitting. Coordinates from the edge of the slices were selected manually from the standard image and fitted with a circle. We reconstructed the curved images with Bresenham’s line algorithm into straight images considering that electrodes passing by the same radius belong to the same column in the straightened image (Figure 2D). The straightened images were used for further analysis.

#### Light Responses

Voltage signals of 200 ms before light onset were considered as baseline. An electrode is considered as detecting a light response if the maximum depolarizing voltage is greater than 15 standard deviations from the baseline signal. If an electrode recorded a light response with the mentioned standard, we define the electrode here as an active electrode (Figure 2A). For measuring signal propagation distance, we first measured the furthest two active electrodes in the *x*-axis in a straightened image. Assuming that the signals always propagated symmetrically to both sides, we divided the measured value by two as the distance to the stimulus center. The amplitude of the light response is defined as the maximum recorded voltage amplitude. Peak latency is the time latency to reach 80% of the maximum voltage amplitude, and the offset latency is defined as when the recorded voltage reached half of the maximum amplitude at the repolarization phase (Figures 2A,B—timepoints 2, 3, and 4). Significance tests were performed with the Wilcoxon rank-sum test.

#### Linear Function Fitting

We fitted the peak latency and the offset latency with linear function to get the kinetic of the light response. We first extracted the peak latency and offset latency from INL and averaged the latencies from the same column (to take the INL as one thin layer). From each slice under each stimulus condition and duration, we acquired data of either peak latency or offset latency to distance away from the stimulus (Figure 6E). Then, we fitted the traces with a generalized Gaussian distribution as shown below and found the optimal beta value for different conditions.

$$G\,\left(x;\beta \right)=\frac{\text{exp}\,\left(-{\left|x\right|}^{\beta }\right)}{2\,\,\left(1+1\,/\,\beta \right)}$$

β = 3.6 for peak latency of control and MFA group, β = 2.5 for peak latency of Cx36 knock-out group, and β = 6.7 for offset latency of control, MFA, and Cx36 knock-out groups. After confirming β for the different conditions, we fitted the linear functions only to the group of 160 ms duration because of best fitting results. Only the samples that showed a high fit quality (*r*^2^ > 0.65) were included to the results. After fitting, we defined the mean ± 1.5 standard deviation of distance as the proximal (central) area and the electrodes located further as the distal area for each individual slice. Linear functions are then fitted to the proximal and the distal points separately.

The analysis of vertical slices including statistical analysis (Wilcoxon rank-sum test) was performed using custom-written Matlab code (MathWorks Inc., Natick, MA, United States).

### Data Analysis—Flat-Mount Retina

All the recordings were first filtered using a 100-Hz high-pass filter and a 3-kHz low-pass filter and spike sorted using the CMOS MEA Tool software (Multichannel Systems MCS GmbH, Reutlingen, Germany). The sorting software is based on a convoluted independent component analysis algorithm (Leibig et al., 2016). The identified units were post-processed manually to remove false positive units or redundant units. Then, the RGCs were ordered by the distance to the light stimulus. The spiking of RGCs is displayed as raster plot. For robust light response detection, we compared the spike number 200 ms before and 200 ms after light onset for 12 repeats and ran a Student’s *t*-test. When the spiking activities were significantly different (*p* < 0.05), the RGC is considered to have a robust light response.

## Results

### Electrical Imaging the Signal Propagation in Vertical Slices

To investigate light-induced signal propagation across and within different retinal layers, the sliced retina was placed onto the CMOS MEA in a vertical fashion (Figure 1 and “Materials and Methods” section).

After light stimulation was projected onto the PR layer *via* a mirror, we recorded the light-induced LFP from the slices (Figure 2A). Electrical recording by all electrodes is visualized in a color code. Here positive extracellular voltages, which indicate cell hyperpolarization, are coded in red, while negative extracellular voltages that indicate cell depolarization are coded in blue. This process of visualizing signal propagation *via* voltage changes over time is called electrical imaging (Figure 2B; Zeck et al., 2017). In Figure 2B, we selected five instances in time as demonstration: light onset (1), depolarizing phase (2), peak of depolarization (3), repolarizing (4), and finishing (5). Among the five instances, the voltage at time points (2), (3), and (4) were used for quantifying the recorded signals and their dynamics (definition given in the “Materials and Methods” section—light response).

It is important for this study to confirm that the recorded LFPs induced by light stimulation match the slice dimension. We therefore stained the slice using Hoechst nuclear staining and overlaid with the color-coded voltage signal induced by light (Figure 2C). The three major retinal layers are clearly visible and allow matching the recorded voltages. To confirm that the recorded signals represent light-induced LFPs, we performed an additional pharmacological experiment, where inhibition of ionotrophic glutamate receptors strongly reduces the signal amplitude in the IPL and the GCL as compared to the control experiment (Supplementary Figure 2).

To quantify signal propagation, electrical images from the curved slices were reconstructed and straightened (Figure 2D, see “Materials and Methods” section). After straightening, each column of electrodes represents a functional unit including all cell layers from PRs to RGCs. The vertical signal transduction pathway within one column is revealed after a stimulus is given (Figure 2E). Notably, signals underneath the photoreceptor layer show a positive polarity, while below the other layers we detected negative extracellular voltages. This result is expected, considering that light onset hyperpolarizes PRs and horizontal cells and depolarizes the majority of all other retinal cells. It also proves that the light-induced voltage changes can be assigned broadly to the photoreceptor layer or the inner retinal layers. We cannot exclude return currents from bipolar cell dendrites to the positive extracellular potentials, which, however, does not affect the following analysis.

In the following discussion, we focus on the negative voltage deflections (depolarizing signal) to study the signal propagation in lateral direction in the retinal slice. We introduced a threshold for the recorded LFP on each individual electrode. An electrode will only be evaluated if the recorded signal exceeded the threshold (Figure 2A, see “Materials and Methods” section for definition). Electrodes recording supra-threshold signals are referred to as an active electrode in the following discussion.

Using the presented methodology, we now analyze the signal propagation for different light stimuli.

### Signal Propagation in a Retinal Slice Depends on the Duration and the Background of the Activating Light Stimulus

**FIGURE 3:**
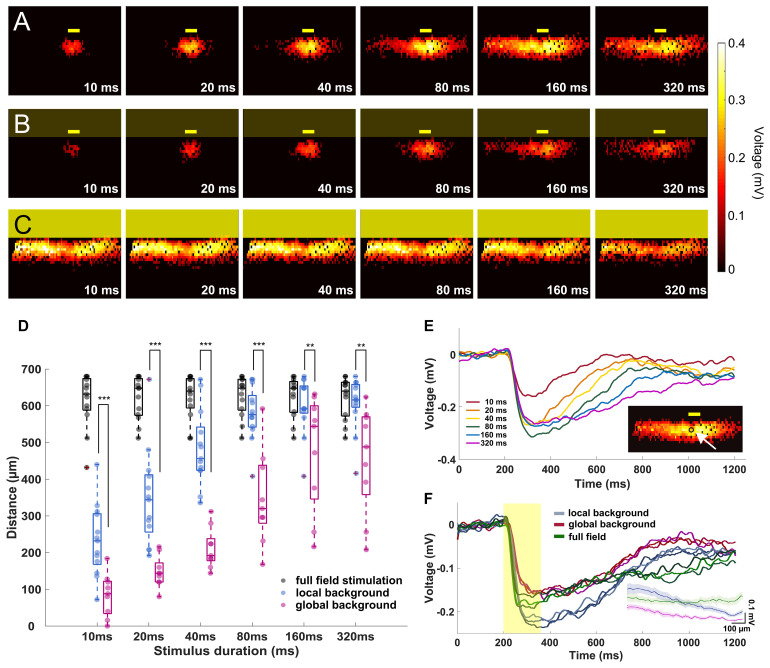
The signal propagation distance increases with stimulation duration. **(A–C)** Heat maps showing active electrodes for different stimulus durations. The stimulus durations range between 10 ms (left) and 320 ms (right). The heat map color represents the amplitude of the extracellular peak voltage. Yellow boxes mark the light stimulus. Size of the light stimulus: 100 μm wide × 30 μm high. **(A)** Local background stimulus. **(B)** Global background stimulus. **(C)** Full field stimulus. **(D)** Evaluation of the signal propagation distance for different light stimulus conditions. The distance increases with stimulation duration for both local (*n* = 13 slices) and global background stimuli (*n* = 9 slices). For all stimulus durations, the signal propagated significantly further under local background (no surround inhibition) than for global background (with surround inhibition). Significance tests were performed with the Wilcoxon rank-sum test. **P < 0.01 and ***P < 0.001). Under full-field stimulation (*n* = 13 slices, black symbols), there was no difference between the stimulus durations. **(E)** Extracellular voltages recorded from an electrode locates downstream of the local stimulus with local background from different stimulus durations. The stimulation duration does not affect the voltage amplitude but only the duration of the responses except for 10 ms. In the insert, the arrow points to the electrode from which the signals are shown. **(F)** Overlaid extracellular voltages recorded from three electrodes downstream of the stimulus area with local background (blue lines), global background (red lines), and full-field stimuli (green). The yellow box represents the 160-ms light pulse. The voltage decreased when stimulation was performed on global background or using full-field stimulus, suggesting the recruitment of an inhibitory input. The insert shows the average peak amplitude *versus* distance from the stimulus center for the local background stimuli, global background stimuli, and full-field stimuli.

Next, we evaluate the signal propagation using the active electrodes under three different light stimulus conditions: (i) stimulus presented on a local background, (ii) stimulus presented on a global background that activates the inhibitory surround to the small-field stimulus, and (iii) a full-field stimulus that served as control to confirm the homogenous attachment and the extracellular recording of the slice on the electrode array (Figures 1E, 3A–C; see “Materials and Methods” section).

In the first experiment, we investigated the signal propagation upon presentation of local light stimuli of six different durations. We found that not only the signals propagated much further in lateral direction than the size of the stimulus, but also the propagation distance was stimulus duration dependent (Figures 3A,D; see Supplementary Videos V1, V2). As the stimulus duration increased from 10 to 320 ms, the mean propagation distance increased from 233 to 607 μm (*n* = 13 slices) and saturated after a certain distance (around 600 μm). The saturation most likely occurred because the signal has reached the border of the electrode array (∼500 μm from the stimulus center). Note that the slice curvature allows for the measurement of maximal propagation distance larger than 500 μm. However, for longer stimuli like 160 and 320 ms, the size of the sensor array could lead to an underestimation of the real propagation distance.

To further investigate if the wide lateral signal propagation would be suppressed by surround inhibition, we provided the small-field stimulation on a global, low-intensity background stimulus. Indeed, now the light-induced extracellular signal was restricted to smaller areas, with mean distances of 85 μm when stimulated with 10 ms light pulse and 460 μm when stimulated with 320 ms light pulse (Figure 3D, *n* = 9 slices). The propagation distance was significantly smaller under global background illumination compared to local background illumination, irrespective of the stimulus duration (Wilcoxon rank-sum test, Supplementary Video V3). Two effects shall be distinguished here: (1) the propagation distance reduced significantly compared to the local background condition, however, (2) the trend of increasing propagation distance with increasing stimulus duration remained. The last result indicates that the duration dependent effect of lateral signal propagation is independent of the stimulus background.

To confirm whether the observed effect of stimulus duration dependent propagation was caused by different signal amplitudes, we examined the raw traces from one active electrode right downstream of the stimulus area for different stimulus durations (Figure 3E). The light evoked similar voltages except for 10 ms, showing that the duration does not affect the voltage intensity of the light-induced responses.

The reduced propagation distance in global background condition as compared to local background condition may be explained by a reduced signal amplitude. The signals recorded from three electrodes underneath the stimulus of either local or global background (both from 160 ms stimulus duration) suggest that the central amplitude is inhibited by activation of inhibitory surround (Figure 3F).

When the entire slice was stimulated with the full field stimulation, the signal was detected over a distance of between 605 to 620 μm (*n* = 13 slices) regardless of the stimulus duration, which corresponds to the maximum distance of the electrodes covered by slices (Figures 3C,D, also see Supplementary Video V4). For the full-field stimulus, a reduced amplitude as compared to local stimulus on local background was detected, which strengthens the hypothesis of recruitment of the inhibitory surround (Figure 3F).

Our results suggest that global background stimuli not only reduce the amplitude of stimulated activity but also reduce the distance signal propagated laterally. Lateral signal propagation distance is duration dependent regardless of background stimulus. This may imply that the further the depolarizing retinal cells are located from the stimulus, the longer stimulus durations are required to activate these cells. Both findings may have implications on the spiking activity of RGCs.

### Remote Modulation of Light-Induced RGC Activity Depends on Stimulus Duration and Background

**FIGURE 4:**
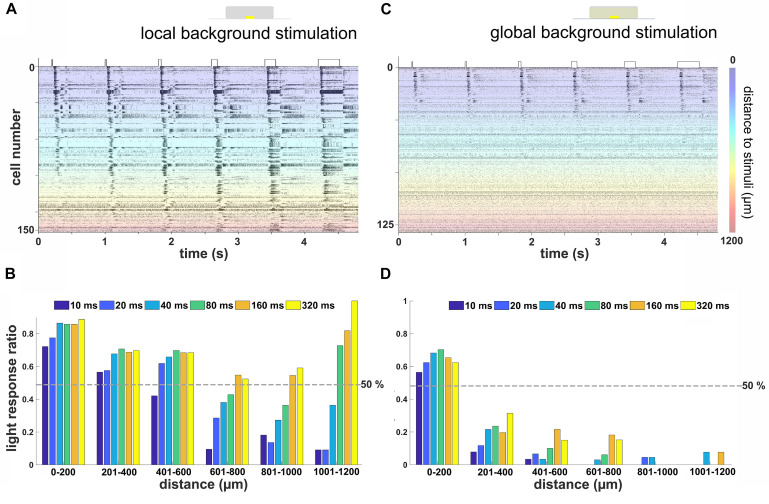
Remote activation in retinal ganglion cells (RGCs) from local light stimulus is background and duration dependent. **(A)** Raster plot of RGCs from one flat-mounted retina stimulated with local stimuli of durations on a local background. The *Y*-axis marks the number of cells in one retina, each of them stimulated 12 times. The color codes for the distance of the RGC from the local stimulus. Most of the RGCs located close to the stimulus (blue-green area) were activated by all stimulus durations, while the RGCs located at the far distance (orange-red area) only responded to relatively longer stimuli. **(B)** Ratio of RGCs with robust light response for different stimulus durations on local background *versus* distance to stimulus center (*n* = 385 RGCs from three retinas). **(C)** Rasterplot of RGCs from the same flat-mounted retina shown in **(A)** with stimulation on a global background. Most RGCs located close to the light stimulus show light responses, while those located distally were not activated. The scale bar marks the distance of the RGC from the stimulus center. **(D)** Similar evaluation as in **(B)** for stimulation on a global background. Few RGCs located further than 200 μm away from the stimulus showed a robust light response (*n* = 281 RGCs from three retinas).

The results obtained in the vertical slice raised the question if increasing the duration of a spatially localized light stimulus affects the spiking activity from more distal RGCs in both local and global background stimulus conditions. To answer the question, we repeated the same light stimuli (Figure 1E) using flat-mount retinas. A representative raster plot showing the spiking activity of 153 identified RGCs to 12 repeats of each stimulus duration is shown in Figure 4A. In the local background condition, more than half of the RGCs located within 400 μm from the stimulus were light responsive regardless of the stimulus duration. A 10 ms stimulus failed to evoke light responses in more than half of the RGCs located further than 400 μm from the stimulus. At a distance larger than 600 μm, only the stimuli longer than 160 ms were able to evoke light responses in the majority of RGCs (Figures 4A,B).

This result showed that, for a local stimulus without a global background, the further the RGC is located from the light stimulus, the longer stimuli were required to evoke light responses. This fits qualitatively with our previous finding on the vertical slice and suggests that the LFP propagation distance translated into spiking activity of RGCs at remote stimulus locations. Note that the increase in light response ratio for long stimuli at distances further than 1,000 μm from the stimulus center (Figure 4B) is caused by the low number of detected cells and may not have any mechanistic basis.

However, when an additional global background stimulus was presented, only nearby RGCs were activated. The raster plot of the spiking activity from 126 RGCs spiking to 12 repeats identified in one retina exemplifies this finding (Figure 4C, same retina as in Figure 4A). Within a 200-μm distance, more than half of all recorded RGCs showed a robust light response irrespective of the stimulus duration (Figure 4D). Surprisingly, longer stimuli such as 160 or 320 ms failed to evoke a robust light response in most of the RGCs located further than 200 μm away.

This result differs from the previous finding in vertical slice that the propagation distance increases with the stimulation duration regardless of the background illumination. We therefore hypothesize that the lateral propagation analyzed and presented in Figure 3 does not reach the RGC layer. Therefore, we went back to the vertical slice and analyzed the signal propagation in more detail.

### Global Background Stimulus Prevents Signal Propagation to the Ganglion Cell Layer in the Distal Area

To understand why we detected the lateral signal propagation over a long distance in the vertical slice but failed to confirm the result at the level of RGC spiking activity, we re-analyzed the electrical imaging results from vertical slice recordings by assigning the active electrodes to different retinal layers. Considering that each slice has a slightly different thickness, the layers were assigned by the relative position of the electrode to the corresponding depth of the slice based on the study of Ferguson et al. (2013). We divided the retinal layers as follows: the OPL occupies 18% of the slice, the inner nuclear layer (INL) takes 26%, and the rest of 56% was assigned to IPL + GCL. We further selected from IPL + GCL the last three rows (48 μm) and assigned them to GCL only. This tentative separation may contain sublamina 5 of IPL, which cannot be resolved here. Based on nuclear staining of the cell layers (Figure 2C), we evaluated a subset of slices (*n* = 5). Although the division calculated from our staining was slightly different (OPL: 13%, INL: 23%, and IPL + GCL: 64%), this would translate to only one electrode difference for individual layers and was not applied in the following.

**FIGURE 5:**
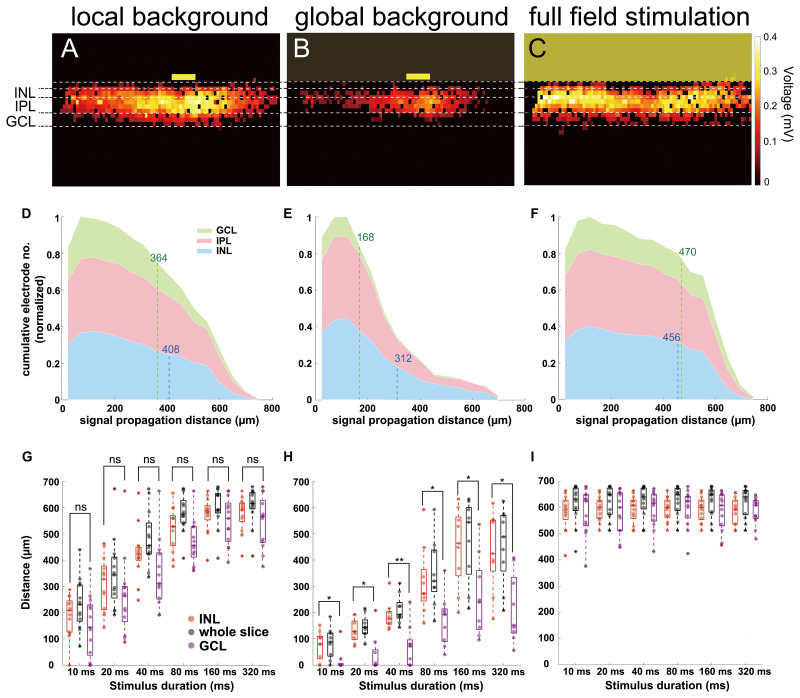
Signal propagation in the inner nuclear layer (INL) and ganglion cell layer (GCL) in three light stimulus conditions. **(A–C)** Heat maps showing active electrodes from 160 ms light-induced field potential. The position of different retinal layers is marked. The heat map color codes for the maximum extracellular voltage. **(A)** Local background stimulation. **(B)** Global background stimulation. **(C)** Full-field stimulation. The yellow boxes mark the stimulus position and size [100-μm wide in **(A,B)**]. **(D–F)** Cumulative distribution diagrams from three different stimuli. The diagrams present the normalized active electrode number to distance from the stimulus in different retinal layers under 160 ms light stimulus. **(D)** Local background stimulus. **(E)** Global background stimulus. **(F)** Full-field stimulus. Numbers labeled at the INL (blue dashed lines) and at the GCL (green dashed lines) mark the 80% limit in the distribution as a benchmark distance to avoid the misjudgment of the propagation by outlier electrodes. **(G)** Box plot of signal propagation distance under local background stimulus measured in whole slice (black), INL (red), and GCL (purple). The signal propagation distances in INL and GCL show no significant difference in any of the stimulus duration. Each symbol represents the result from one slice under the specified condition. **(H)** Same as **(G)** for global background stimulus. The signal propagation distances are significantly larger in INL than in GCL. **(I)** Same as **(G)** for full-field stimulus; the signal propagation distances are similar regardless of measuring the whole slice, INL, or GCL. Significance tests were performed with the Wilcoxon rank-sum test. *P < 0.05 and **P < 0.01.

When the layer separation following (Ferguson et al., 2013) was applied to the electrical slice images, we found that signals propagated from INL to GCL homogeneously with full-field stimulation (Figure 5C) and was slightly decreased in the GCL for local background stimulation (Figure 5A). For a local light stimulus on a global background, however, distal signals were mainly detected in the INL but not in the GCL (Figure 5B). We use cumulative distribution diagrams to present the homogeneity of signal propagation in different retinal layers (Figures 5D–F). If the signal propagates homogeneously through the lateral and the vertical directions, then the proportion of active electrodes in the INL and in the GCL would be similar relative to the number of electrodes in each layer. If signals are confined to INL and do not propagate to GCL, a clear difference is expected. The example diagrams (Figures 5D–F) comprise the cumulative number of active electrodes in different retinal layers from all the recorded slices (13 from local background and full field, 9 from global background) stimulated with 160 ms light pulse. The signal traveled 86% further in INL than in GCL under global background stimulus (312 μm in INL and 168 μm in GCL, Figure 5E) as compared to only 12% under local background condition (408 μm in INL and 364 μm in GCL, Figure 5D) and almost no change under full-field stimulus as expected (456 μm in INL and 470 μm in GCL, Figure 5F).

The same conclusion can be obtained by examining the lateral signal propagation distances in different layers separately: the signal in INL propagated significantly further than in the GCL upon stimulation with a global background for all stimulus durations (*n* = 9 slices).

For local background or full-field stimulus conditions, the differences of propagation distances in INL and GCL showed no significance (Figures 5G–I, *n* = 13 slices). These results explain why we could not record RGC activity at a remote distance (>200 μm) from the stimulus if a global background was presented.

The failure of LFP propagation to the RGC layer under a global background condition may be explained by the low peak amplitudes in the INL. To understand if the signal amplitude in INL would affect the signal detection in the GCL, we compared the average peak voltage amplitudes in INL under the two stimulus conditions (local background and global background). The peak amplitude decreases with distance for the local background stimulus. A similar trend is observed for the small-field stimulus on global background, albeit with a smaller starting amplitude (Supplementary Figures 3A,B).

Indeed when relating the peak amplitude in the INL to the peak amplitude in the GCL (considering electrodes in the same column), a positive linear correlation is detected (average correlation coefficient = 0.8, *n* = 9 slices), suggesting that the signal amplitude in the GCL is highly correlated to the amplitude in the INL (Supplementary Figures 3C,D). On the other hand, there was little correlation under full-field stimulation (average correlation coefficient = 0.4, *n* = 9 slices, Supplementary Figure 3E).

By considering electrodes not covered by the slice (i.e., electrode iii in Figure 2A), we obtained the basic noise level with “amplitudes” below ∼50 μV (Supplementary Figure 3F). When comparing the signal amplitudes in INL under local background and global background stimuli, more electrodes covered by the slice detected signals below 50 μV in the global background stimulus condition and thus did not pass the threshold for being considered as an active electrode (Supplementary Figures 3C–E). These findings suggest that local background stimulation could evoke a higher signal amplitude; therefore, more electrodes would detect signals higher than ∼50 μV as compared to the global background condition, resulting in a longer signal propagation distance.

Although the separation in individual layers (Figure 5) explains our finding in the flat-mounted retina (Figure 4), it remains unclear which retinal circuitries might be involved for this effect. We therefore investigated the kinetics of the light responses by using the peak latency and the offset latency of the extracellular voltage from the recorded signals (Figure 2A, for definitions, see “Materials and Methods” section).

**FIGURE 6:**
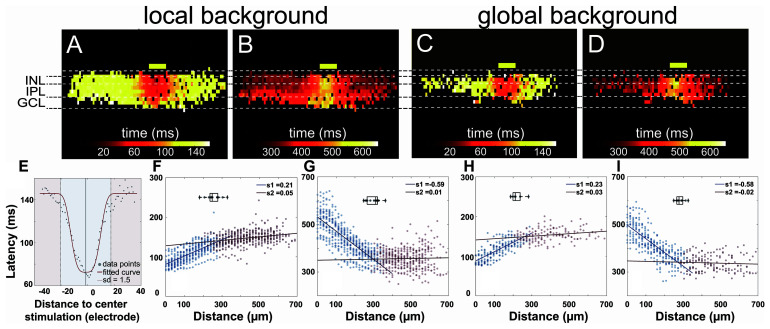
Proximal and distal areas show two different light response kinetics. **(A–D)** Heat maps of active electrodes with the color code representing peak latency **(A,C)** and offset latency **(B,D)** in different retinal layers upon 160-ms light stimulus. **(A,B)** Local background stimulus. **(C,D)** Global background stimulus. The yellow boxes mark the stimulus position and size (100-μm wide). **(E)** Exemplary scatter plot overlayed with the fitted curve. Peak latencies to distance collected from the inner nuclear layer (INL) in a single slice can be fitted with a normalized Gaussian distribution (see also the “Materials and Methods” section). The area from the stimulus center to a distance of 1.5 standard deviation is considered as proximal area (blue); the further area is considered as distal area (red) for each individual slice. **(F–I)** Scatter plots of latency *versus* distance to stimulus from active electrodes in INL from multiple slices overlaid with fitted linear regression. **(F)** Peak latency for local background stimulus. **(G)** Offset latency for local background stimulus. **(H)** Peak latency for global background stimulus. **(I)** Offset latency for global background stimulus. Points in two areas were fitted with linear regression separately. The boxplots in each subplot show the average distances for the proximal areas. The average distances for the individual plots are 255 μm **(F)**, 299 μm **(G)**, 221 μm **(H)**, and 283 μm **(I)**.

It is worth mentioning that the peak latencies we measured in slices start at around 80 ms (Figure 6), recorded by the most proximal electrodes. This value is longer than the first-spike latencies of RGCs recorded in flat-mount preparations, which are, on average, ∼60 ms (Supplementary Figures 4–5). While the RGC first-spike latencies are in agreement with previous work (Stutzki et al., 2014; Tengölics et al., 2019), the discrepancy between ∼80 and ∼60 ms originates from the fact that RGC spikes occur earlier than the light-induced LFP in the flat-mount preparation (Supplementary Figure 6) and the different ways we evaluated the latency. However, it does not influence our further analysis.

The heat maps of peak latency and offset latency from local and global background stimuli both show two very different phases from proximal and distal areas relative to the light stimulus (Figures 6A–D). To define the proximal and the distal area, we first extracted from each electrode in the INL the peak latency and the offset latency. We fitted the latencies *versus* distance using a normalized Gaussian distribution (Figure 6E, see “Materials and Methods” section) to the same slices analyzed in Figure 5 and defined the division between proximal and distal area at the point where the standard deviation = 1.5. The average proximal sizes were 255 and 299 μm, measured from the stimulus center for peak latency and offset latency in the local background stimulus (*n* = 13 slices), and 221 and 283 μm for peak latency and offset latency, respectively, in the global background stimulus (*n* = 9 slices).

After identifying the proximal and the distal areas, we fitted linear regressions to the data in these two areas separately and obtained two distinct slopes that indicate the signal propagation velocity (Figures 6F–I). The slopes show two phases of the signal propagation: (1) a first phase with low propagation speed (“slow phase”) confined to the proximal area and (2) a second phase with high propagation speed (“fast phase”) which occurs in the distal area.

For the peak latency, the slopes in the first phase for the local and the global background stimuli are 0.21 and 0.23, respectively, translating to the signal propagation speed of 4.8 and 4.3 μm/ms, respectively. For the second phase, the slopes are 0.05 and 0.03 from the local and the global background stimuli, respectively, translating to a propagation speed of 20 and 33 μm/ms. The most likely candidate for the fast signal propagation may be gap junctions. In a flat-mount preparation, gap junction-mediated propagation of field potentials has been estimated to propagate with velocities between 5 and 20 μm/ms (Menzler and Zeck, 2011).

The separation of signal propagation in two phases is clearly seen in the offset latency as well: the slopes of -0.59 and -0.58 in the proximal area and 0.01 and -0.02 in the distal area under local and global background stimuli, respectively, suggested that there are two signal transduction mechanisms in the slices.

The analysis of signal kinetics showed that the INL receives and processes signals from upstream even though the signals are not always transduced to the GCL and that the light responses in the proximal and the distal areas are mediated by two different, slow and fast, mechanisms. In the following, we investigate the hypothesis that the fast signaling within the INL is mediated by gap junctions.

### The Fast Signal Propagation in the INL Is Mediated by Gap Junctions

**FIGURE 7:**
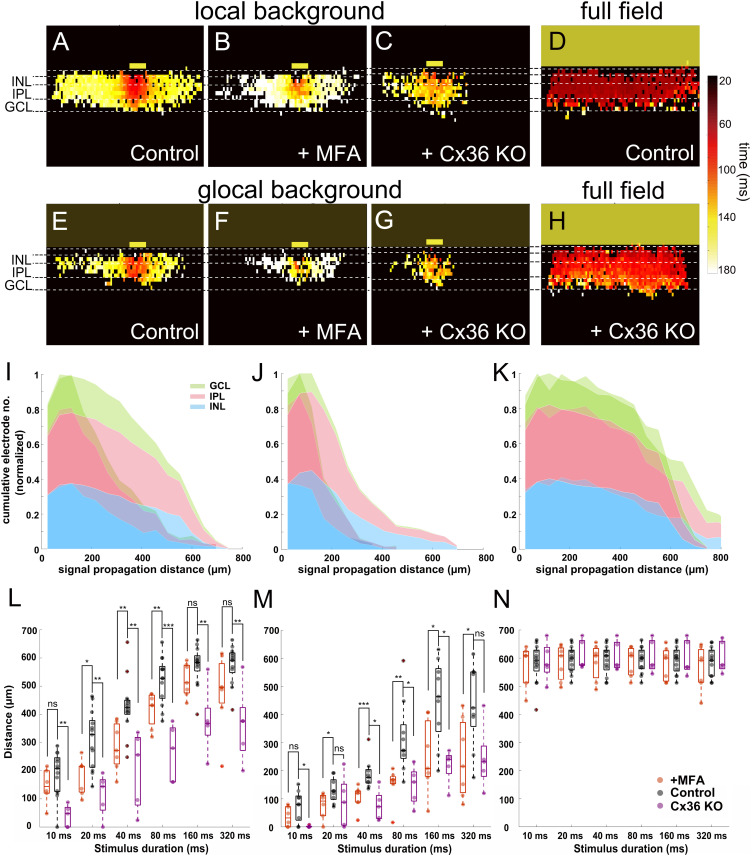
Blockage of gap junctions inhibit the lateral signal propagation. **(A–H)** Heat maps of active electrodes with the color code representing peak latency in different retinal layers stimulated with 160-ms light pulse. **(A)** Local background stimulus. **(B)** Local background stimulus + MFA (100 μM). **(C)** Local background stimulus from Cx36 KO slice. **(D)** Full-field stimulus. **(E)** Global background stimulus. **(F)** Global background stimulus + MFA. **(G)** Global background stimulus from Cx36 KO slice. **(H)** Full-field stimulus from Cx36 KO slice. The yellow boxes mark the stimulus positions. **(I–K)** Overlaid cumulative distribution diagrams from Cx36 KO slices (saturate color) with WT control slices (transparent color) under three different stimuli. The diagrams present the normalized number of active electrodes *versus* the distance from stimulus in different retinal layers (160 ms light stimulus). **(I)** Local background stimulus. **(J)** Global background stimulus. **(K)** Full-field stimulus. **(L)** Box plot of signal propagation distances under local background stimulus measured in INL with control (black), +MFA (red), and Cx36 KO (purple). The signal propagation distances after adding MFA or from the Cx36 KO slice reduced significantly in most of the stimulus durations. Each symbol represents the result from one slice under the specified condition. **(M)** Same as **(L)** for global background stimulus. The signal propagation distances decrease after adding MFA or from Cx36 KO slice compared to the control group. **(N)** Same as **(L)** for full-field stimulus; the signal propagation distances are similar regardless of measuring from the control, MFA, or Cx36 KO slices. Significance tests were performed with the Wilcoxon rank-sum test. *P < 0.05; **P < 0.01; and ***P < 0.001.

Our previous results showed that the light response recorded in slices can be divided into two different phases, with a second phase characterized by fast propagation, which may be mediated by gap junctions. To test this hypothesis, we applied MFA (100 μM) to block gap junctions. After MFA application, the propagation distance in INL decreased under both local and global background stimulus conditions (*n* = 7 slices). Color-coded peak latency heat maps show the effect for a local background stimulus with reduced signal propagation distance (Figures 7A,B). The same effect was shown when the stimulus was provided on a global background (Figures 7E,F), indicating that gap junctions indeed mediate the lateral signal propagation that we recorded.

Among all gap junctions, Connexin 36 (Cx36) is the most commonly encountered one and plays an important role in the retina (Veruki et al., 2010; Trenholm and Awatramani, 2017). We therefore asked if and how Cx36 itself would affect the lateral signal propagation. To answer this question, we applied the same stimuli to slices obtained from Cx36 knock-out (Cx36 KO) mice (Meyer et al., 2014; Tetenborg et al., 2019). The signal propagation distances in the Cx36 KO slices for both local and global background stimuli for all durations significantly decreased in most cases (Figures 7C,G,L,M; see also Supplementary Video V5; *n* = 5 slices). The full-field stimulus on both MFA applied (*n* = 7 slices) or Cx36 KO (Supplementary Video V6, *n* = 5 slices) slices showed almost no difference to the propagation distance, proving that the decrease is not caused by any preparation artifact (Figures 7D,H,N). The cumulative distribution diagrams from Cx36 KO slices once again show the loss of active electrodes at the distal area after the loss function of Cx36 gap junctions (Figures 7I–K). These results suggest that when the retina is stimulated with light, the lateral signal transduction in the INL is strongly dependent on the gap junctions, predominantly from Cx36.

## Discussion

### Long-Range Lateral Signal Transduction

The first interesting result refers to the wide propagation of light-induced signals in vertical slices, which extends by far the classical receptive field of BCs (Berntson and Taylor, 2000) or RGCs (Farrow and Masland, 2011; Baden et al., 2016).

Secondly, under local background and full-field stimulation, the signals traveled to the RGC layer, indicating that the signals that we recorded in INL were very likely the mixture of BCs and ACs since BCs are the only cell type in INL that give excitatory input to RGCs. In the condition with local stimulus on a global background, the signal did not travel to the RGC layer at the distal area, suggesting that the signal recorded at the distal area was most likely attributed to ACs. Even if there could be a contribution from the excitatory postsynaptic potential from BCs, it would be minor because the amplitude is not even enough to evoke RGC spiking activity. The failure to evoke distal RGC spiking activity is most probably related to the low LFP peak amplitudes under this experimental condition (see Supplementary Figure 3).

Previous studies showed that the responses of BCs (Franke et al., 2017) and of RGCs (Sagdullaev and McCall, 2005) decrease when stimulated with large spots as compared to small spot stimulation (i.e., the size of central stimulation). Our results are in line with these reports in that, when an inhibitory background illumination was applied (the global background), the light response amplitude decreased (Figure 3F). One possible explanation could be the lateral inhibition from horizontal cells. In our preparation, however, it is not possible to evaluate the contribution from horizontal cells because they are located at the transition of the LFP polarity change (from hyperpolarization to depolarization). We observed that, with global background illumination, signal propagation was restricted in the RGC layer to the proximal area (Figures 4, 5). One recent study showed that central stimulation suppresses the distal response evaluated in flat-mounted retina at the ganglion cell population response (Deny et al., 2017). This explains why when there is a global background illumination, the RGCs at the distal area were unable to be activated. To the distal cells, their central receptive field was already activated by the background illumination, therefore not able to respond to their distal stimulation, which is the small-field stimulation.

However, signal propagation in INL is always detected regardless of the background condition (Figure 5). After blocking gap junctions or by measuring Cx36 knock-out mice, the significantly reduced lateral propagation distance in INL implies that Cx36 is involved in the mechanism and plays an important role in lateral signal propagation (Figure 7).

Cx36 gap junction couplings are known to be expressed between PRs in OPL and between All-BC, All-All, GC-AC, and GC-GC in the IPL (Bloomfield and Volgyi, 2009; Trenholm and Awatramani, 2017). The short-range gap junction coupling between cones has been found to improve the contrast sensitivity by increasing the signal-to-noise ratio at the cost of losing some visual acuity by 0.5 cone diameters (DeVries et al., 2002). However, the 0.5 cone diameters will not explain the far distance of signal propagation that we observed from our experiments.

**FIGURE 8:**
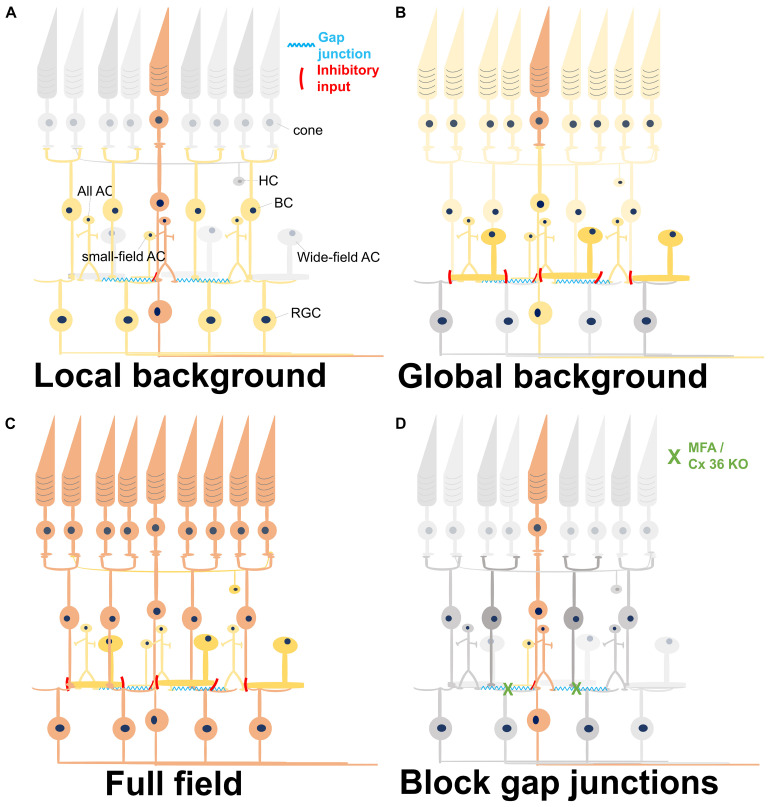
Summary of light-induced signal transduction pathways under different conditions. Different colors represent the level of activity. Gray, no activation; light yellow, weak activation; dark yellow, medium activation; orange-red, and strong activation. **(A)** A local stimulus strongly activates the central photoreceptors, which excite the synaptically connected bipolar cells (BCs). The BCs activate all amacrine cells *via* gap junctions, eventually forming a network activation. Small-field amacrine cells (ACs) may also be activated by the local light stimulus and give a weak inhibitory input to the BCs. The network in the inner retina leads to a broad activation of remote retinal ganglion cells (RGCs). HC, horizontal cell. **(B)** Weak activation of all photoreceptors by a weak global background in addition to the strong activation of central photoreceptors by a local stimulus. Except for the local circuitries, the wide-field inhibitory ACs are also recruited by the global background, weakening the responses of BCs and RGCs activated by the strong local stimulus. **(C)** Full-field stimulation activates all photoreceptors underneath the stimulus area and also their downstream BCs. In this case, all the excitatory and the inhibitory retinal networks are activated, with excitation being stronger than inhibition. **(D)** Reduction of gap junctional coupling leads to a local activation of PRs and BCs, but not to a wide network activation. Therefore, the activity is confined to a restricted area.

Our results of reduced signal propagation are restricted to the INL, therefore most likely to be mediated by Cx36 between All-BC, All-All, or GC-AC. Lateral signal propagation has been reported in whole-mount preparations upon blocking inhibition to BCs (Toychiev et al., 2013) and were also abolished by blocking gap junctions. The underlying mechanisms reported here and by Toychiev et al. (2013) appear similar: inhibition restricts the gap junction-mediated tangential signal propagation. Our explanation of gap junctions in the INL being the main driver for the fast signal propagation phase implies that, first, light-induced signals require to be processed by bipolar cells (Figure 8A). This leads to a constant time delay (Figures 6F,H). Gap junctional coupling has been reported across species with different expression patterns (Kovacs-Öller et al., 2017). Further examination of our hypothesis may therefore involve interspecies comparison.

All ACs function over six to seven log units of intensity (Xin and Bloomfield, 1999). Though the most well-known function of AII ACs is to convey the light-induced signal from rod pathway to cone BCs and RGCs under scotopic light intensity, evidences in mice (Pang et al., 2007), rabbit (Bloomfield et al., 1997), and primate (Strettoi et al., 2018) have shown that All ACs also work at photopic range of light. Since signal propagation through gap junctions is bidirectional (Veruki et al., 2010), All ACs would also be activated with photopic stimulation *via* the activation of ON-cone BC (Manookin et al., 2008; van Wyk et al., 2009; Hartveit and Veruki, 2012). We hypothesize that the long-range field potentials originate from the local stimulation of cones; these cones activate the ON-cone BCs in the central area, which further activate the connected All ACs. Because these All ACs are connected further to cone BCs, a network activation and thus a longer distance of signal propagation is conceivable (Figure 8A). The All-ON CBC-All network has been reported in the photoreceptor-degenerated retina (Margolis et al., 2014; Trenholm and Awatramani, 2015), proving their strong connectivity.

### Inhibitory Network in the Inner Retina Under Different Light Conditions

The results obtained from global background stimulation showed that, though signals did not propagate to the RGC layer, we did record depolarizing activity in the INL. As argued before, these activities are most likely contributed from ACs.

Previous studies have shown that the concept of receptive field may be much more complex than the classical center-surround concept. OFF RGCs respond to distal stimuli by the disinhibition of glycinergic ACs *via* GABAergic ACs (Deny et al., 2017). Large-field light stimulation was found to suppress BC inhibition with serial connections between inhibitory ACs. More specifically, wide-field GABAergic ACs disinhibit BCs *via* inhibiting GABAergic ACs through GABA_*A*_ receptors (Eggers and Lukasiewicz, 2010). On the other hand, disinhibition of the GABAergic network by glycinergic ACs was also reported (Franke et al., 2017). Crossover inhibition among ACs showed the complex modulation of inhibitory input to BCs (Molnar and Werblin, 2007; Hsueh et al., 2008).

The main difference between the two stimuli investigated here in depth (i.e., stimulus with local or global background) is that local stimulation would only stimulate small-field ACs which are involved in local inhibitory circuitries. Global background includes the activation of wide-field ACs that provide GABAergic input to BC axon terminals (Franke and Baden, 2017) and inhibit BC activity. When using a full-field strong stimulus instead of low-intensity global background, the BC depolarization overcomes the inhibitory input and activates the downstream RGCs. The proposed signaling based on our results is summarized in Figure 8. Even though wide-field stimulation was found to activate serial connections and further suppress the inhibitory postsynaptic currents in BCs (Eggers and Lukasiewicz, 2010), our results (Figure 3) and previous reports (Franke et al., 2017) both showed that the inhibition is much stronger than the disinhibition effect, eventually causing the decrease of BC response.

### Stimulus Duration-Dependent Signal Propagation

In all of the results from slices, one phenomenon that was independent from background condition or drug application was the stimulus-dependent signal propagation. The longer the stimulation, the further the LFP propagated. Patch-clamp recording from BCs showed that only the light intensity but not the duration changes the amplitude or the latency of the transmembrane voltage (Euler and Masland, 2000). This is in line with our recording, where except for the very short stimuli (10-ms duration), all other stimuli evoked extracellular signals with very similar amplitude and latency (Figure 3E). Therefore, the duration-dependent effect is not caused by the decay of the extracellular voltage from cells at the light stimulus.

Signals recorded by distal electrodes imply that more depolarizing cells are activated by longer stimuli. While very short light pulses could simply seem like noisy signals to the retina, longer stimulus not only could mean a more important signal but also more chances for post-synaptic cells to get enough input from their dendrites and send signals further downstream. Note that the longest duration that we used was 320 ms; therefore, it is not in the same range for adaptation (seconds to minutes).

Increasing the activated area and thereby the number of activated retinal cells could mean that the important input signal is amplified and could also reduce the visual acuity and lead to an inaccurate inference of the stimulus position. Though longer stimuli did activate more cells in the visual processing, it only turns into RGCs spiking output when there is no background stimulation (Figure 4). This interesting result could mean that, when there is no other visual stimulation in the environment, RGCs choose to sense anything that they can detect, even the stimulus located far away from their own classical receptive field. Future research may evaluate this hypothesis.

### Electrical Imaging Signal Propagation With High-Density CMOS MEA

In this study, we demonstrated how to analyze signal (LFP) propagation across and within different retinal layers in vertical slices using high-density CMOS-based MEAs.

Electrical imaging of LFPs using CMOS-based MEAs has been applied before to study other brain areas such as the well-known tri-synaptic hippocampal formation (Hutzler et al., 2006; Ferrea et al., 2012) or cortical structures (Viventi et al., 2012; Wickham et al., 2020) with the focus on epileptiform activities. Examples of propagating LFP were shown along the hippocampal CA region (Channappa et al., 2014) and in photoreceptor-degenerated flat-mounted mouse retina (Menzler and Zeck, 2011). Electrical imaging at a coarser spatial scale discussed the possibility of non-synaptic propagation of epileptiform activity in the unfolded hippocampus (Choi et al., 2014).

In retina research, among the first results revealed by electrical imaging, the developing retina of the RGC layer were the retinal waves (Meister et al., 1991). Recently, electrical imaging at high spatial–temporal resolution using CMOS-based MEAs revealed shrinkage of these waves during ontogeny down to the size of the spatial receptive fields of RGCs (Maccione et al., 2014). Whereas in the healthy retina the synchronous retinal output largely disappears, it is consistently detected in photoreceptor-degenerated retinas (Menzler and Zeck, 2011; Menzler et al., 2014). However, the RGC spiking alone does not provide a complete description of the functional changes occurring in these retinas. Strong sub-threshold oscillations of transmembrane potential (Choi et al., 2014; Menzler et al., 2014) lead to LFPs, which emerge spontaneously and propagate at different speeds across the retinal layers.

Electrical imaging upon light stimulation may be affected by the CMOS MEAs sensitivity (Bertotti et al., 2017). Here we avoided any light artifact using a 45° mirror next to the sensor array and projected the light as shown in Figure 1. We further demonstrate that there are no light artifacts (i.e., Figure 2A, trace iii; Figures 7F,G), which may interfere with our results.

One limitation of the current study is the failure to detect both LFPs and single-cell activity of RGCs and potentially of spiking BCs (Baden et al., 2013) in the vertical slice. This may be overcome by adding three-dimensional electrodes (Jones et al., 2020) to the sensor area and thereby enabling a tight contact to the slice. A second caveat is the mixture of signals from ON and OFF bipolar cells. Future work may combine two-photon imaging of the two major inner retinal layers (Zhao et al., 2020) with CMOS MEA recording (Lee et al., 2018). Alternatively, calcium imaging within the restricted layers (INL and GCL) of the retinal slices may investigate to what degree the observed effect of remote activation (Figure 4) is cell class specific.

## Conclusion

Electrical imaging light-induced signal propagation in different retinal layers visualizes how signals propagate within and across the distinct retinal layers. We applied the method for one simple, pulsatile, and localized stimulus and analyzed the conditions of remote activation. Future work may extend the approach to more elaborate stimuli to reveal the full potential of the intriguing signal processor implemented by the mammalian retina.

## Data Availability Statement

All datasets presented in this study are included in the article/Supplementary Material.

## Author Contributions

M-JL and GZ designed the study. M-JL performed the tissue preparation, CMOS MEA recording, and data analysis. Both authors shared the task of writing the manuscript.

## Conflict of Interest

The authors declare that the research was conducted in the absence of any commercial or financial relationships that could be construed as a potential conflict of interest.
