# Supplementary material for: Electrical Imaging of Light-Induced Signals Across and Within Retinal Layers
Source: Front Neurosci. 2020 Nov 19;14:563964. doi: 10.3389/fnins.2020.563964 (PMC7717958; doi:10.3389/fnins.2020.563964)
Supplement: Supplementary file 7 [file Data_Sheet_1.pdf]

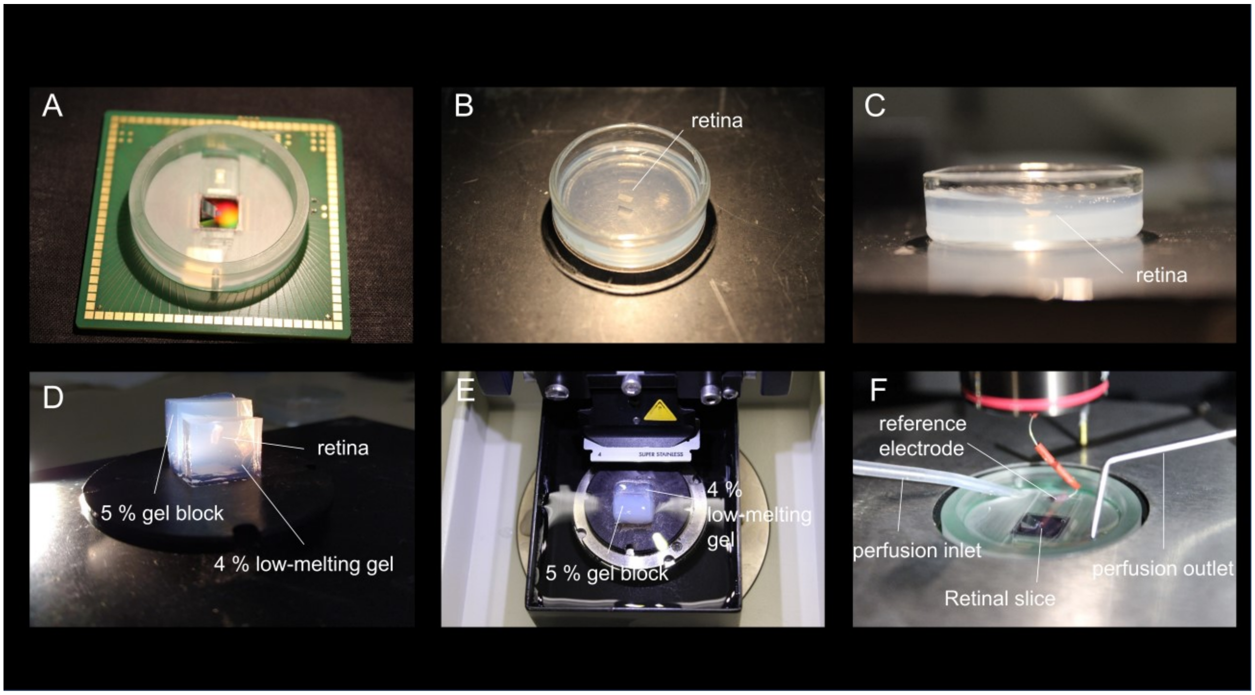

Supplementary Figure 1: Pictures of slice preparation on CMOS MEA. **(A)** A CMOS MEA with a 45 degree mirror adapt on top. **(B)** Dissected retina embedded in the 4% low-melting agarose gel. **(C)** Side view of dissected retina embedded in the 4% low-melting agarose gel. **(D)** A 5 % broad range agarose gel block glued right next to the gel block with retina on the specimen disc. **(E)** Flat-mount retina with gel blocks in buffer tray filled with Ames' medium on vibratome. **(F)** Retinal slice on CMOS MEA in the recording amplifier. Perfusion tubings are marked as well as the external Ag/AgCl reference electrode.

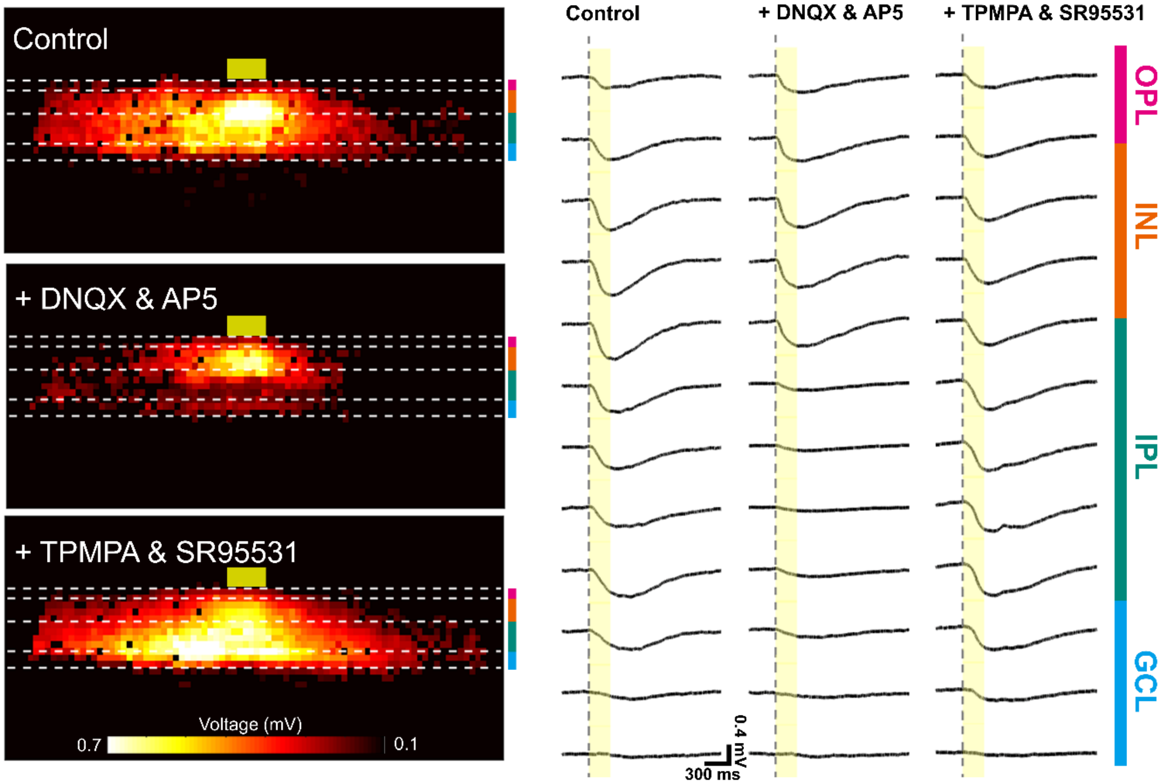

Supplementary Figure 2: Light responses modified by pharmacological treatments in retinal slices. Left: Heatmaps of light response signal amplitudes across different layers when stimulated with 160 ms "local background" stimulus in control condition (top), with DNQX and AP5 (middle) and with TPMPA and SR95531(bottom). Colorbars correspond to the same colorbars at the right hand side, which label their layers. Yellow boxes represent the given light stimulus (100µm x 30 µm). Right: Signal traces averaged from 12 stimulus repeats from one column of electrodes right below the light stimulus.

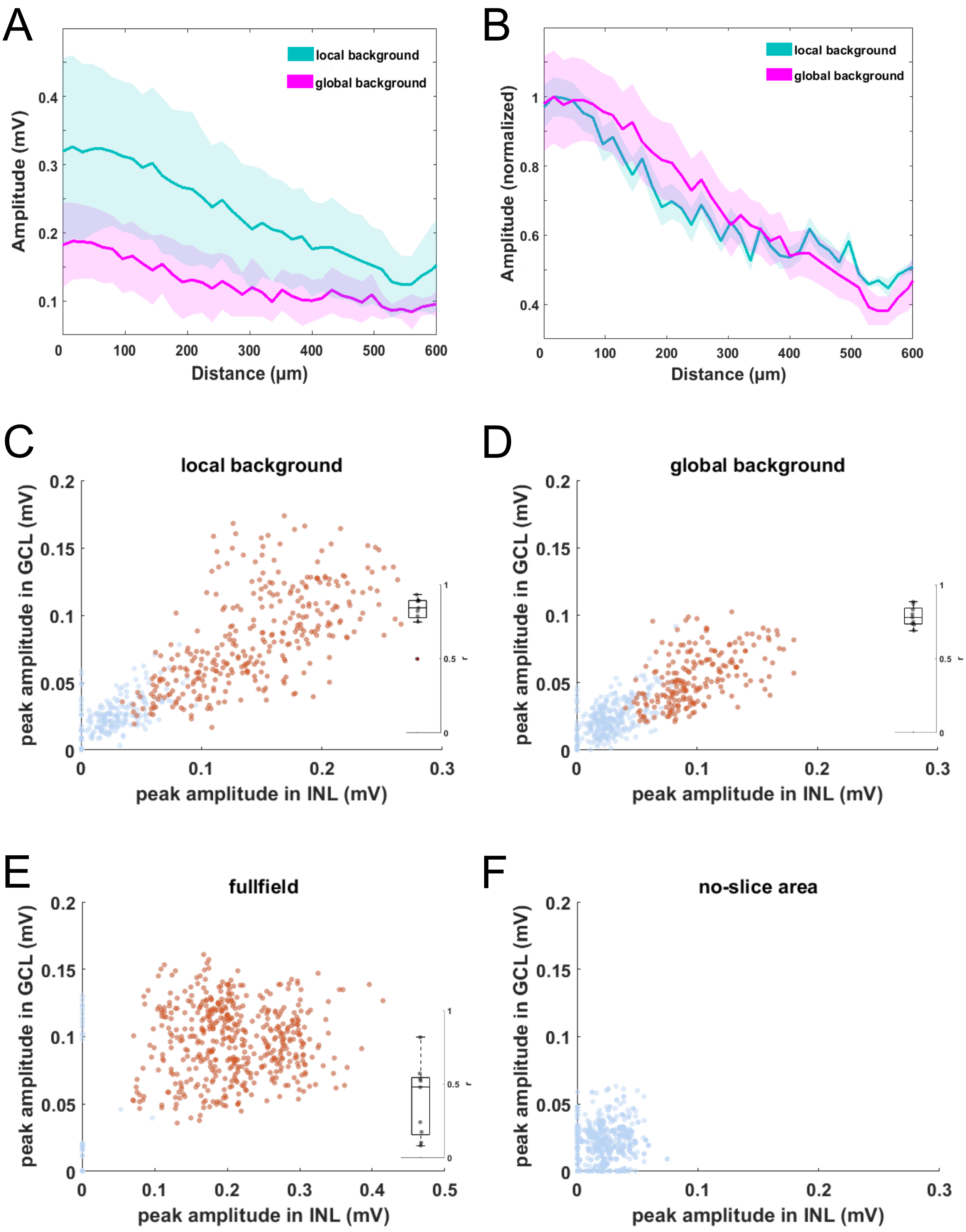
A
local background
global background
Amplitude (mV)
0.4
0.3
0.2
0.1
0
100
200
300
400
500
600
Distance (μm)
B
local background
global background
Amplitude (normalized)
1
0.8
0.6
0.4
0
100
200
300
400
500
600
Distance (μm)
C
local background
peak amplitude in GCL (mV)
0.2
0.15
0.1
0.05
0
0.1
0.2
0.3
peak amplitude in INL (mV)
D
global background
peak amplitude in GCL (mV)
0.2
0.15
0.1
0.05
0
0.1
0.2
0.3
peak amplitude in INL (mV)
E
fullfield
peak amplitude in GCL (mV)
0.2
0.15
0.1
0.05
0
0.1
0.2
0.3
0.4
0.5
peak amplitude in INL (mV)
F
no-slice area
peak amplitude in GCL (mV)
0.2
0.15
0.1
0.05
0
0.1
0.2
0.3
peak amplitude in INL (mV)

Supplementary Figure 3: Stimulus background condition affects the peak amplitude of extracellular signals but not the decaying tendency. **(A)** Averaged peak amplitude versus distance recorded in INL under local and global background stimulus conditions. Local background stimulus evoked higher amplitude than global background stimulus. In both conditions the amplitude decreases with distance from stimulus center. **(B)** The normalized signal amplitudes show that the tendency for amplitude decrease by distance is irrelevant to background condition. This indicates that the diminished distal GCL activity in the global background condition is relevant to the evoked amplitude. **(C-D)** Scatter plots of averaged maximum peak amplitudes in INL vs averaged peak amplitudes in GCL from the same column from one single slice. Blue dots represent electrodes that were not considered as active electrodes, red dots stand for active electrodes. Box plots at the right bottom corner show the correlation coefficient values from 9 slices in different light stimulation conditions. For both local and global background stimuli, the signal amplitude in GCL is positively correlated to the signal recorded in the INL ($r = 0.81 \pm 0.12$ for local background **(C)** ; $r = 0.79 \pm 0.06$ for global background **(D)**, r = Pearson correlation coefficient, Mean ± SD). This indicates that for localized light stimulation, the signal amplitude in the GCL is highly correlated to the signal amplitude from its upstream INL regardless of the background stimuli.

(E) Scatter plot of averaged maximum peak amplitudes in INL vs averaged peak amplitudes in GCL for full field stimulation. The correlation decreases to $r = 0.4 \pm 0.24$

(F) Signals from two electrode rows outside the retinal slice were selected to present the basic noise level, which is around 50 μV.

These figures clearly show that though the low amplitude signals might be missed with the active electrode threshold, however, the pattern of scatter plots from non-active electrodes in local and global background stimulation conditions (blue dots) are very similar to the pattern where there is no retinal neurons ("no-slice" area). This figure strongly suggests that the threshold for active electrodes used in this study was reasonable.

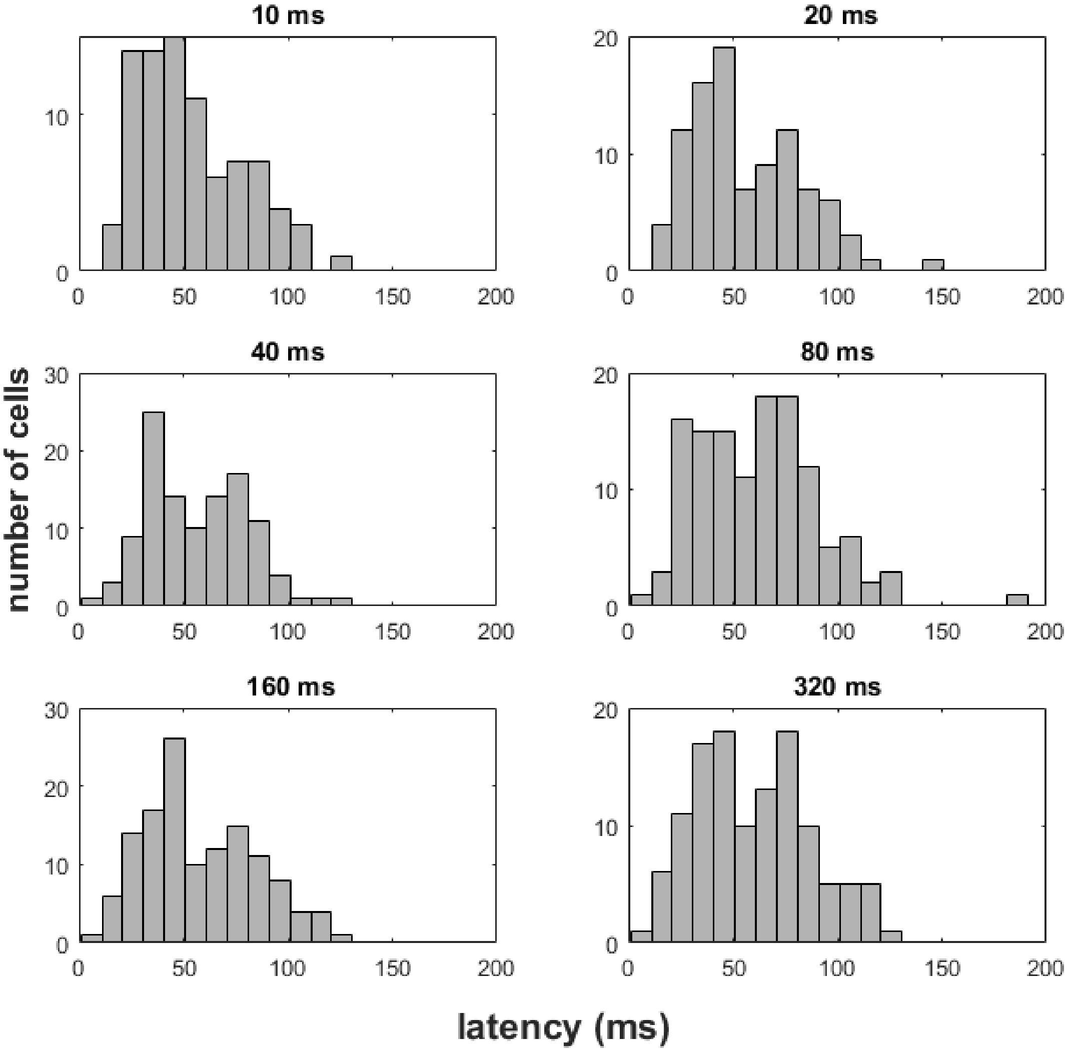

Supplementary Figure 4: Mean latency of the ON RGCs in different stimulus durations for flat-mount recording. Histograms show light responsive ON RGCs latency vs distance distribution in different stimulus duration under local background stimulation. Most of the RGCs have the latencies between 20-100 ms. The average latency for each duration are 10 ms= 55.1, sd=26.7; 20 ms= 57.7, sd=28.9; 40 ms= 58.5, sd=26.5; 80 ms= 63.3, sd=31.8; 160 ms= 58.7, sd=27.9; 320 ms= 60.6, sd=28.5. unit= ms. Mean ± SEM. n=158 cells from 3 retinas.

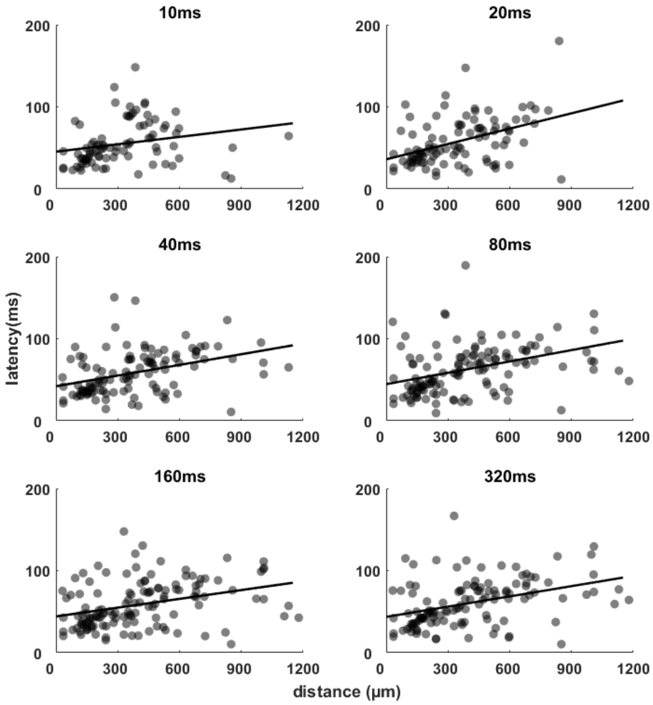

Supplementary Figure 5: Scatter plots of first spike latency vs distance in ON RGCs in local background stimulation with different stimulus durations. The lines are the linear regression fittings of the scatter plots. The response latency increases with the distance from the stimulus center in all stimulus durations.

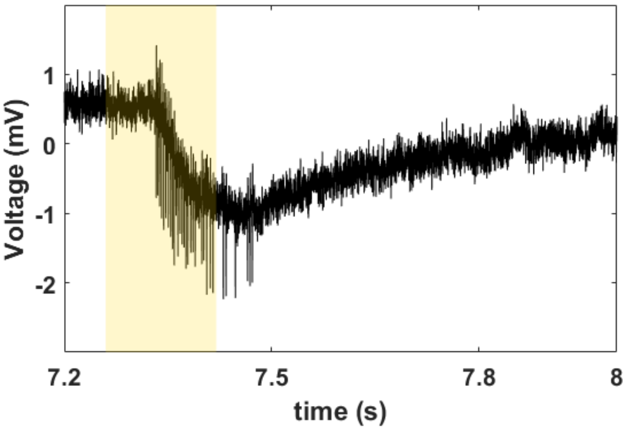

Supplementary Figure 6: Raw trace of one electrode recording the RGC spiking activity with 160 ms local background stimulus. Yellow box represents the light stimulus. The spiking occurs earlier than the LFP peak.
